# The Institutional Library

**Published:** 1915-03-06

**Authors:** 


### THE INSTITUTIONAL LIBRARY.

### Handbook for Orderlies.

A Nursing Manual for Nurses and Nursing Orderlies. By Duncan C. L. Fitzwilliams, M.D., F.R.C.S. (London: Henry Frowde, and Hodder and Stoughton. 1914. Pp. 466 + viii. Price 6s. net.)

This is a useful manual, and its appearance at the present date is appropriate; such a remark is perhaps specially true of the sections dealing with bandaging and with first-aid subjects, in which, for obvious reasons, there is at present a widespread interest. Mr. Fitzwilliams' book, however, is not one merely created for an emergency. It is intended as a manual of general nursing, and it is well planned for the purpose at which it aims. The chapters on anatomy and physiology are sufficient without being over-elaborate, and the advice and directions given are associated with reasons based upon knowledge. Altogether the book is admirably suited for the constituency to which it appeals. If we may dissent from the author on a single point it would be in the directions given for the treatment of various diseases. It is no part of a nursing orderly's duty to hold an opinion on the most suitable stage for operation in appendicitis or to select remedies for the treatment of ringworm. Short, dogmatic, and unqualified statements on such matters are apt to encourage a profession of knowledge which is neither seemly nor justified. The old tag which encouraged the shoemaker to stick to his last illustrates a principle which ought to be sternly applied by those who aspire to teach nurses the true sphere of their duty. With this exception we find ourselves in general agreement with Mr. Fitzwilliams' pages, and have pleasure in recognising the lucid and careful style in which his book is written.

### A Sanitary Inspector's Manual.

Students' Text-book of Hygiene. By W. James Wilson, M.D., D.Sc., D.P.H., Bacteriologist to the Counties of Down and Antrim, Lecturer in Hygiene and Public Health, Queen's University, Belfast. (London: William Heinemann. 1915. Price 8s. 6d.)

It is not a matter for surprise that a new students' text-book of hygiene, containing but 263 pages and (though printed upon somewhat thick paper) weighing less than a pound and a half, should prove to be a manual rather than a comprehensive treatise. Dr. James Wilson, in his preface, modestly defines the intended scope of usefulness of his book. He has kept his object well in view, and has compressed and epitomised so well that he is able not only to divide his work into twenty chapters, but to include, amongst the subjects of these, heredity and eugenics, personal hygiene, and tropical diseases. The subjects, whilst necessarily dealt with in a terse and introductory way, embrace so wide a range as to contain brief references to climatology and meteorology, artificial lighting, the disposal of the dead, and even cooley itch and the danger of ill-kept swimming-baths. The information is excellent, and the omissions noticeable are few, though "certain experiments" in connection with ventilation receive scant notice, and no acknowledgment is made to the eminent physiologist who conducted them; we find, too, no mention of the spray method of disinfection. The proof-reading has been carefully done (though "prostatis" for "prostatitis" has remained), and a fairly full index is given. The illustrations are well reproduced, and we suggest with diffidence that a wiser selection of subjects for them might have been made.

The popular aim of the book is exemplified by such passages as: "The unfortunate circumstance about the decline" (of the birth-rate) "is that it is due to the relative infertility of the most valuable stocks in the race. . . . To-day, by our social legislation, we are annulling the effects of a selective death-rate, and our only hope of salvation lies in a selective birth-rate." Again, by the "friends and relatives of the deceased . . . the horrors of corruption are not contemplated. . . . The advocates of cremation think with disgust of the slow process of decomposition in contrast to the rapid and purifying action of fire."

Written in a simple, fluent style, with bold type usefully employed to facilitate reference, the book affords remarkably easy and agreeable reading. It is a work which, whilst it will be welcomed by medical officers of health, will also appeal not only to students of medicine, but to students of sociology and (as the author suggests) "to teachers, sanitary inspectors, health visitors, and all who are interested in public health"; for such readers it is, by its style and diction, admirably adapted.

### Modern Hæmatology.

Clinical Examination of the Blood and its Technique. A Manual for Students and Practitioners. By Professor A. Pappenheim. Translated and adapted by R. Donaldson, M.A., F.R.C.S. Edin., D.P.H. (Bristol: John Wright and Sons, Ltd. 1914. Price 3s. 6d.)

Hæmatology has become indeed a proud and stiff-necked science; amateurs are warned off by the increasing complexity of the technical terminology and by the doubts they must entertain as to the durability of the inferences to be drawn from the cytology of a film. One may cordially agree with the translator in acknowledging that the clinical examination of the blood should be more frequently carried out, but it is distinctly unfair to reproach the practitioner. The technique is certainly not difficult as clinical laboratory work goes nowadays, but except for the examination of the film, the processes have to be carried out almost immediately. There is no sending of a small tube of blood to the nearest laboratory and waiting for a report as for a Widal or Wassermann test. The methods usually employed necessitate the patient being sent to the laboratory. It would undoubtedly be a good thing if the practitioner were to realise how comparatively simple it would be to set up the necessary apparatus for examining the blood in a corner of his study. A little practice, and the whole business becomes rapid and easy. Professor Pappenheim's little book is one which is likely to discourage a beginner at hæmatology; he would find the descriptions of the cells overpoweringly complete, but would miss many of those little practical details of manipulation, attention to which is essential if good films and reliable enumerations are to be obtained. A couple of diagrams are badly needed on page 2 to make clear the way in which the author means the films to be made. In the chapter on counting, the arithmetical procedures could be explained very much more simply, and the same applies to the very cumbersome method of working out the hæmoglobin index—a figure which can be obtained in a trice. On the other hand, more help might have been given in explaining the pitfalls of hæmocytometry. The defects of this book from the beginner's point of view do not prevent due acknowledgment being made to Dr. Donaldson for the excellence of his translation; it is only suggested that he might have "adapted" it a little more. Its Teutonic character would stand leavening with a barm of practical details and an elementary glossary. The two coloured plates of stained cells are beautifully reproduced and very helpful, but the explanatory notes facing the plates need rearranging.

